# Notes and Notices

**Published:** 1898-03

**Authors:** 


### NOTES AND NOTICES.

DR. ALICE B. CAMPBELL will remove her office, after April 1st, to No. 552 McDonough Street, Brooklyn, New York, between Ralph and Patchen Avenues. Telephone, Bushwick 97. Office hours: 9 to 11 A. M., 6 to 7.30 P. M.; none Saturday or Sunday evenings.

ANNUAL REUNION OF THE ALUMNI ASSOCIATION OF THE HAHNEMANN MEDICAL COLLEGE, PHILADELPHIA.—The annual reunion and banquet of the Alumni Association of the Hahnemann Medical College, Philadelphia, will be held on Thursday, May 12th, 1898.

Class reunions will be held at 10 A. M., in Horticultural Hall, Broad Street above Spruce. The business meeting will convene at 4.30 P. M., and the banquet will be held at 7 P. M., at Horticultural Hall.

The Trustees and Faculty of the College extend a cordial invitation to all the members of the Alumni and their friends to attend the fiftieth annual commencement, to be held on the same day, at 2 o'clock, at the Academy of Music, S. W. corner Broad and Locust Streets, Philadelphia.

Banquet cards can be secured by notifying the Secretary. Requests received after Wednesday, May 11th, 1898, cannot be considered. W. D. Carter, M. D., Secretary, 1533 South 15th Street, Philadelphia.

THE AMERICAN MEDICAL PUBLISHERS' ASSOCIATION will hold its fifth annual meeting in Denver, on June 6th, the day preceding the opening of the meeting of the American Medical Association. A liberal attendance is expected, and medical publishers from every portion of America will be present.

CHILDREN'S HOMŒOPATHIC HOSPITAL of Philadelphia, Pa., 926 and 928 North Broad Street. Office of the Chairman, B. W. James, M. D., of the Medical and Surgical Staff, N. E. corner Eighteenth and Green Streets. An examination for two Resident Physicians for the Children's Homœopathic Hospital, of Philadelphia, 926 North Broad St., will be held at the Institution during the first week in May.

Applications should be sent to the Hospital in care of the Medical and Surgical Staff. A large experience is afforded a physician who desires to post himself in general work. Besides the medical and surgical practice of the hospital wards, there is a dispensary of 40,000 applicants annually where clinics for adults and children are held daily. Surgery and outside practice in medicine and obstetrics are also available to the Residents.

